# Dose and blending fraction quantification for adaptive statistical iterative reconstruction based on low‐contrast detectability in abdomen CT

**DOI:** 10.1002/acm2.12813

**Published:** 2020-01-03

**Authors:** Yifang Zhou

**Affiliations:** ^1^ Department of Imaging Imaging Physics Division S. Mark Taper Foundation Imaging Center Cedars‐Sinai Medical Center 8700 Beverly Blvd. Los Angeles California 90048 USA

**Keywords:** adaptive statistical iterative reconstruction, dose reduction, lesion size, minimum detectable contrast, noise correlation

## Abstract

**Purpose:**

The utilization of iterative reconstruction makes it difficult to identify the dose‐noise relationship, resulting in empirical design of scan protocols and inconsistent conclusions on dose reduction for consistent image quality. This study was to quantitatively determine the dose and the blending fraction of adaptive statistical iterative reconstruction (ASIR) based on the specified low‐contrast detectability (LCD).

**Methods:**

A tissue equivalent abdomen phantom and a GE discovery 750 HD computed tomography (CT) were utilized. The normality of the noise distribution was tested at various spatial scales (2.1–9.8 mm) in the presence of ASIR (10–100%) with a wide range of doses (2.24–38 mGy). The statically defined minimum detectable contrast (MDC) was used as the image quality metric. The parametric model decomposed the MDC into two terms: one with and the other without ASIR, each was related to the dose in the form of power law with factors and indices dependent on spatial scales. The parameters were identified by least‐square fitting to the experimental data. By considering the constraint of the blending fraction in the range of [0, 1], the dose and ASIR blending fraction were determined for any specified low‐contrast detectability (LCD), quantified by the MDC at the concerned lesion size.

**Results:**

It was verified that noise distribution is normal in the presence of ASIR. It was also found that the noises obtained from the subtractions of adjacent slices had an underestimate of 20% as compared to the ground truth noises, regardless of the spatial scale, pitch, or ASIR blending fraction. The least‐square fitting for the parametric model resulted in correlation coefficients from 0.905 to 0.996. The root‐mean‐square errors ranged from 1.27% to 7.15%.

**Conclusion:**

The parametric model can be used to form a look‐up‐table for dose and ASIR blending fraction. The dose choices may be substantially limited in some cases depending on the required LCD.

## 1 INTRODUCTION

Iterative reconstruction (IR) has been widely used in computed tomography (CT) as an attempt to reduce patient dose.^1^, ^2^, ^3^, ^4^, ^5^, ^6^ In practice, it is important to first define the target image quality, then plan the CT protocol in a way that the dose consequence is known prior to the scan. However, the application of IR makes the task difficult as the noise‐dose relation from filtered‐backprojection (FBP) is no longer valid. The difficulty makes the protocol design empirical and may lead to different conclusions on whether dose reduction and quality preservation can be both achieved. McCollough et. al. reported a degradation of low‐contrast resolution at the reduced dose levels of 25–50% from the volume CT dose of 16 mGy in a phantom study.^7^ Pooler et. al. reported that aggressive dose reduction (60–70% down to volume CT dose of 5 mGy) with IR resulted in inferior diagnostic performance for liver lesions.^8^ However, Saiprasad et. al.^9^ concluded that higher correct classification rates were achieved with IR even at the volume CT dose as low as 7.6 mGy in a multi‐center multi‐vendor phantom study.

The abovementioned conclusion variations may be attributed to the complex and yet to be discovered relationship of the dose to low‐contrast detectability (LCD) and the iterative reconstructions. Appropriate dose reduction depends on many factors such as the required image quality for the concerned lesion with certain dimension, the IR blending fraction, and the initial dose. In this study, we utilized the LCD as a metric of image quality. The objective was to investigate, for the specified LCD, whether multiple pairs of the dose and IR fraction exist and how they can be determined. Adaptive statistical iterative reconstruction (ASIR) was utilized in the work.

## 2 MATERIALS AND METHODS

### 2.1 Statistically defined minimum detectable contrast

**Figure 1:**
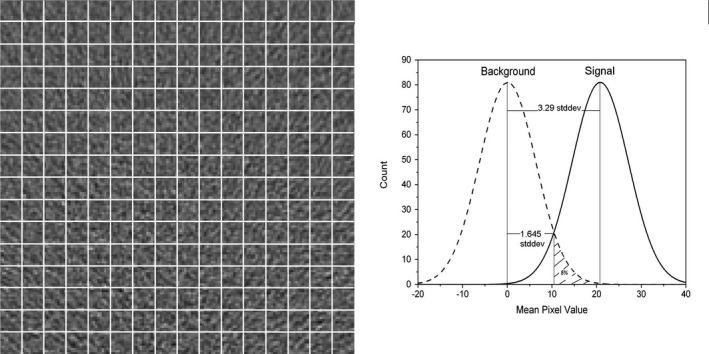
A noise image with a uniform background is partitioned to a matrix (left panel). The distribution of the background mean pixel values from the matrix elements is shown as the dashed curve (right panel). The signal distribution is a shifted curve (in solid). The statistically defined minimum detectable contrast (SD‐MDC) is defined as the shift equal to the standard deviation of either distribution multiplied by 3.29, corresponding to a differentiation confidence of 95%. The SD‐MDC is expressed in percentage in the text.

Because LCD is one of the key aspects in diagnostic CT, the LCD was used as an image quality metric in this study. There have been various studies assessing the LCD in CT.^10^, ^11^ LCD is typically expressed in terms of resolvable contrast (in Hounsfield units or in percentage by normalizing HU by 1,000) for various lesion sizes (thus for various spatial frequencies). We utilized the statistically defined minimum detectable contrast (SD‐MDC). Its concept can be explained as follows: due to the presence of quantum noise in the CT imaging chain, neither the signal (or target) nor the background holds a single constant value. Rather, each contains a statistical distribution. The signal can only be detected from the background if the difference of their mean values exceeds the standard deviation of the distribution by a certain amount. To obtain the statistical distribution, one can make many repeated image acquisitions and quantify the mean pixel fluctuation over time in the area with the dimension of the concerned signal. As a practical alternative, one can also acquire a uniform image and form a matrix by shifting the area of interest across a large uniform region. The statistical distribution is therefore obtained over space using all mean pixel values from the matrix elements. To further illustrate the SD‐MDC, Fig. 1 shows a background image partitioned by a grid on the left side. The noise distribution can be quantified by calculating the mean pixel value from each square cell and plotting the distribution as shown on the right side of Fig. 1. In the presence of the signal, if the noise distribution about the mean value is not altered, the same process can be applied and it results in a shifted distribution as shown in Fig. 1. If the noise distribution follows the Gaussian form, 90% of the distribution is about the distribution mean value within the range of the standard deviation (*stddev*) multiplied by 3.29. The tail on the either side occupies 5%. Therefore, if the signal distribution's mean value differentiates from the background counterpart by 3.29*stddev,* they can be separated with a confidence level of 95%. This threshold difference is defined as SD‐MDC^12^, ^13^]. In order to use the conventional specification of CT contrast in percentage, the SD‐MDC is normalized by 1,000 and expressed in percentage hereafter. As the CT noise distribution varies with the spatial scale, SD‐MDC is also spatial scale dependent.

### 2.2 Quantitative approach

If the SD‐MDC is denoted as α, the spatial scale on which the noise distribution is calculated as *d*, the size specific dose (SSD) as *D*, and IR blending strength (fraction) as *s*, then α can be written as a function of *D*, *d*, and *s* in the following form:

(1) α=α(D,d,s).

We further assume that the dependency on *s* can be separated from the rest and consider that α corresponds to α_0_ when *s* equals zero, then Eq. (1) is written as follows:

(2) $$\alpha \left(D,d,s\right)=\alpha_{0}\left(D,d\right)+\lambda \left(D,d\right)s^{q},$$

where α_0_ and λ are functions of *D* and *d* to be determined, and *q* is a power index to be determined.

We propose that both *α_0_* and λ depend on *D* in the form of power law with the coefficients (*a_0_* and *a_λ_*) and power indices (*b_0_* and *b_λ_*) as functions of *d*, as shown in the following equations:

(3) $$\alpha_{0}\left(D,d\right)=a_{0}\left(d\right)D^{b_{0}\left(d\right)},$$

(4) $$\lambda \left(D,d\right)=a_{\lambda }\left(d\right)D^{b_{\lambda }\left(d\right)}.$$

These equations are based on our previous studies but made more general.^14^, ^15^ We attempted to form a parametric model rather than a derivation from the first principle. Instead of using constant parameters for *b_0_* and *b_λ,_* 14 the dependence of Eqs. (3) and (4) on the spatial scale *d* is considered.^16^ The validity of the above equations depends on the experimental data. The details of the experiment are described in the following section.

Once the explicit form of Eq. (2) is obtained, the IR blending fraction can be expressed in terms of the dose and LCD. The existence of multiple sets of dose and IR blending can be quantitatively analyzed.

### 2.3 Experiment design

A realistically shaped tissue equivalent abdomen phantom (TE‐07, CIRS, Norfolk, VA, USA) was scanned helically with a GE Discovery 750 HD scanner. The phantom dimensions are 25 cm by 32 cm in the cross‐section and 15 cm in the longitudinal direction. The scanner contains a detector system with a full collimation of 40 mm at the iso‐center in the longitudinal direction. Fast kVp switch technology is part of the system design for dual energy acquisitions. The system was equipped with ASIR. The reference SSD was measured at 120 kVp, 200 mAs, and full collimation (40 mm) using a 100‐mm CT chamber (Radcal 10 × 5‐10.3CT, Monrovia, CA, USA). Eleven dose levels were manually chosen in the range of more than tenfold (SSD from 2.24 mGy to 38 mGy at 120 kVp with the clinically used pitch of 1.375). At each dose level, ten ASIR blending fractions (from 10% to 100%) were used to reconstruct the images with the slice thickness of 5 mm and reconstruction diameter of 36 cm. To account for the scatter from the adjacent anatomy, all scans were performed with two other phantoms of similar size at the ends of the studied phantom.

Noise images were extracted by progressively subtracting the adjacent slices in the middle range along the longitudinal direction (seven images from eight interior slices). To investigate whether the adjacent slice subtraction would result in any over or under estimation due to the possible inter‐slice correlation, the ground truth noise images were obtained from subtractions of repeated acquisitions. Two experiments were conducted on different phantoms. First, three repeated image acquisitions were used on the tissue equivalent phantom with the same techniques at various doses (FBP images at the pitch of 1.375 and five SSDs from 13.5 mGy to 23.9 mGy, and at the pitch of 0.984 and 6 SSDs from 4.7 mGy to 36.9 mGy). Second, ten repeated image acquisitions were made on a circular water phantom in diameter of 32 cm with volume CT index of 10 mGy and helical pitches of 1.375 and 0.516. ASIR fractions of 10%, 50%, and 100% were used for reconstructions. The slice width was 5 mm for all images in both experiments. All noise images from subtractions were corrected by a factor of 1/√2.

### 2.4 Normality test and data analysis

All noise images from adjacent slices subtraction were partitioned to four quadrants about the image center. Each quadrant was a square region of 4.2 cm by 4.2 cm. The quadrant was further partitioned into six matrices with element linear dimensions matching as close as possible the lesion sizes of concern: 10 mm, 7 mm, 5 mm, 3.5 mm, 2.5 mm, and 1.8 mm, respectively. Due to the digital truncation from the finite pixel size, the closely matched target sizes turned out to be 9.84 mm, 7.03 mm, 4.92 mm, 3.51 mm, 2.81 mm, and 2.10 mm, respectively. The terms of "lesion size," "matrix element size" or "target size" may be used interchangeably hereafter.

For each noise image and each element size, the average pixel values from all elements in four quadrants formed a distribution. All distributions (N = 4,452 in total due to four missing ASIR image sets among all dose levels) were obtained for six element sizes, eleven doses and ten ASIR blending fraction (10–100%). The distribution normality was examined using Kolmogorov‐Smirnov test (Originlab, MA, USA). Two output parameters were provided from the test: the statistic D delineating the deviation of the cumulative distribution function from the normal distribution, and the p‐value quantifying the significance of the deviation. If the *P*‐value is smaller than the pre‐determined alpha level, for instance 0.05, the normal distribution assumption is rejected.

If the distribution was tested to be normal, the SD‐MDC was computed as the standard deviation of the distribution multiplied by 3.29, indicating the signal can be differentiated from the background with a confidence level of 95%, provided that the SD‐MDC is obtained from the true noise images. To examine the difference between the SD‐MDCs obtained from the adjacent‐slice subtractions and from the inter‐acquisition subtractions that served as the ground‐truth, the SD‐MDC ratios between the two scenarios were calculated at the six afore‐mentioned spatial scales. If the ratios were significantly different from identity as compared to the value's fluctuation, the adjacent‐slice subtraction results were corrected to the ground‐truth. The details of the correction are presented in Section 3.2.

### 2.5 SD‐MDC, ASIR fraction, dose, and lesion size

At each dose level, ASIR fraction and lesion size, the SD‐MDC was averaged from seven noise images obtained from the subtraction of the adjacent interior slices. Correction to the ground truth noise was made if necessary as explained above and elaborated on in Section 3.2. The subsequent analyses were made following the equations in Section 2.2. First, according to Eq. (2), the SD‐MDC results were examined against ASIR fraction for different element sizes (lesion or target sizes of concern) at all dose levels. Least‐square fits were used to find the fitting relation and parameters *α_0_*, λ and *q*. Second, the fitting parameters *α_0_* and λ were further investigated following Eqs. (3) and (4) by checking the power‐law relations to the dose (SSD) using the least‐square fit. Finally, the coefficients (*a_0_* and *a_λ_*) and power indices (*b_0_* and *b_λ_*) were plotted against the element size (lesion size) to find the relations.

### 2.6 Constraint on dose and ASIR blending fraction

From the results in Section 2.5, the ASIR blending fraction can be explicitly expressed as a function of the dose and SD‐MDC at the concerned target size. For the specified SD‐MDC at the target size, the function is dependent on the dose, but is further constrained by the range of [0, 1]. This leads to limited choices of the dose and the ASIR blending fraction. 

## 3 RESULTS

### 3.1 Noises normality test

The results of the normality test from all distributions (N = 4,452) showed that the p‐values are significantly bigger than 0.05, demonstrating the validity of the normal distribution of the noise.

### 3.2 Noises ‐ adjacent slice subtraction versus inter‐acquisition subtraction

**Figure 2:**
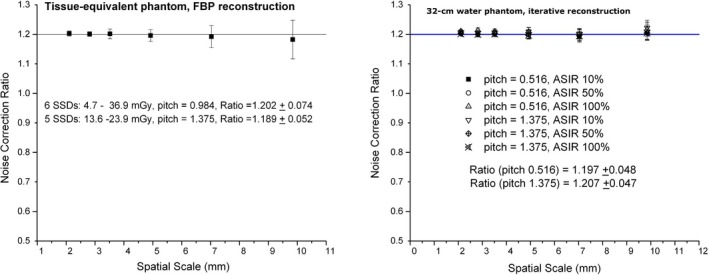
Ratio of the mean pixel value standard deviation from the interacquisition subtractions to the deviation from the adjacent slice subtractions.

Figure 2 shows the results of the ratio of the mean pixel value standard deviation from the inter‐acquisition subtractions to the deviation from the adjacent slice subtractions. At each matrix element size, the ratios among different doses and pitches show a nearly constant value of 1.2 with slight fluctuations (errors ranged from 0.008 to 0.065 with the first experiment, and 0.048 with the second). These findings suggest that the adjacent slice subtraction underestimated the noise and a correction factor of 1.2 should be applied. Regardless of the selections of the phantom, pitch or reconstruction, the results were the same.

### 3.3 SD‐MDC, ASIR fraction, dose, and lesion size

**Figure 3:**
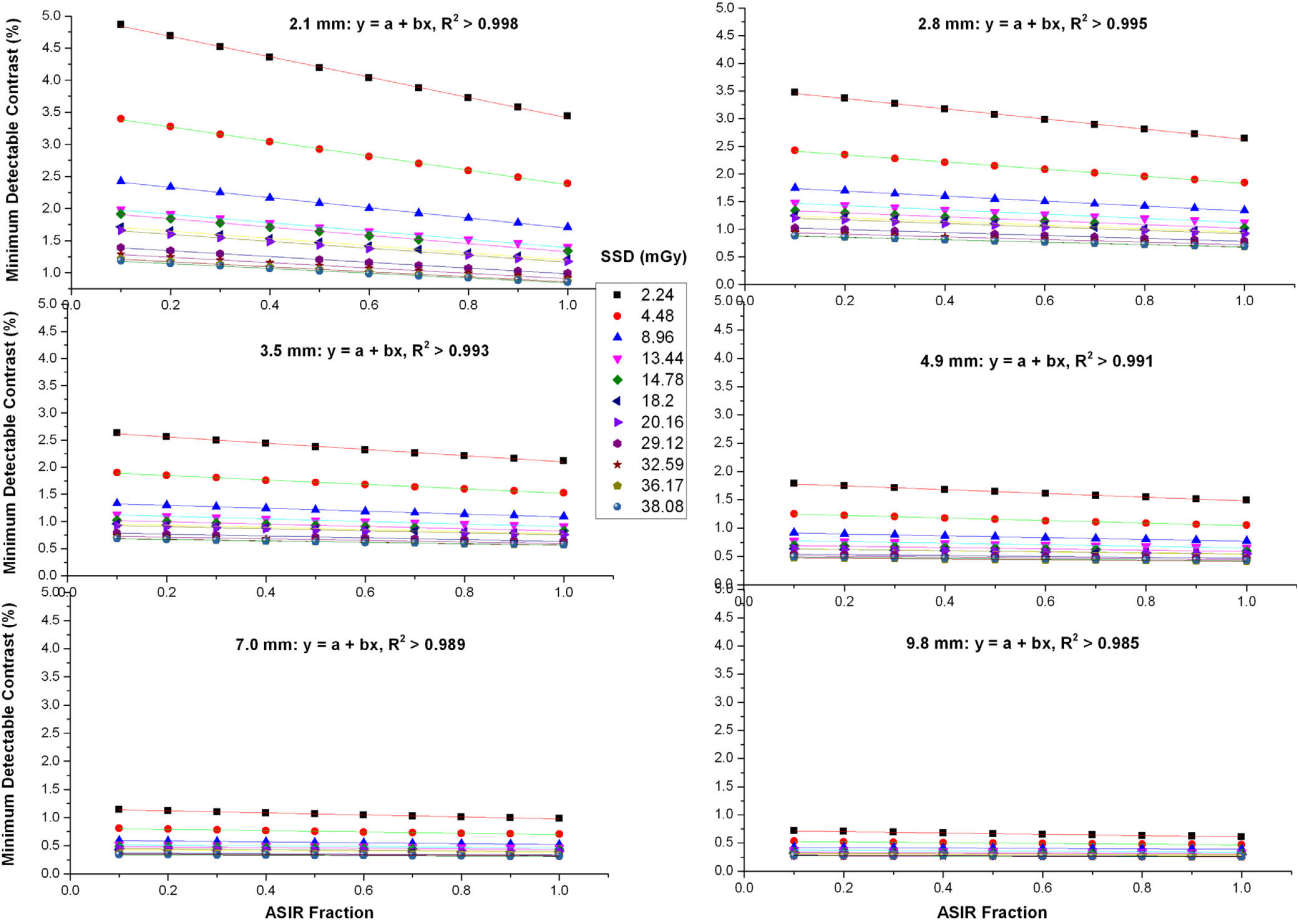
Minimum detectable contrast versus adaptive statistical iterative reconstruction fraction shows linear relationships.

**Figure 4:**
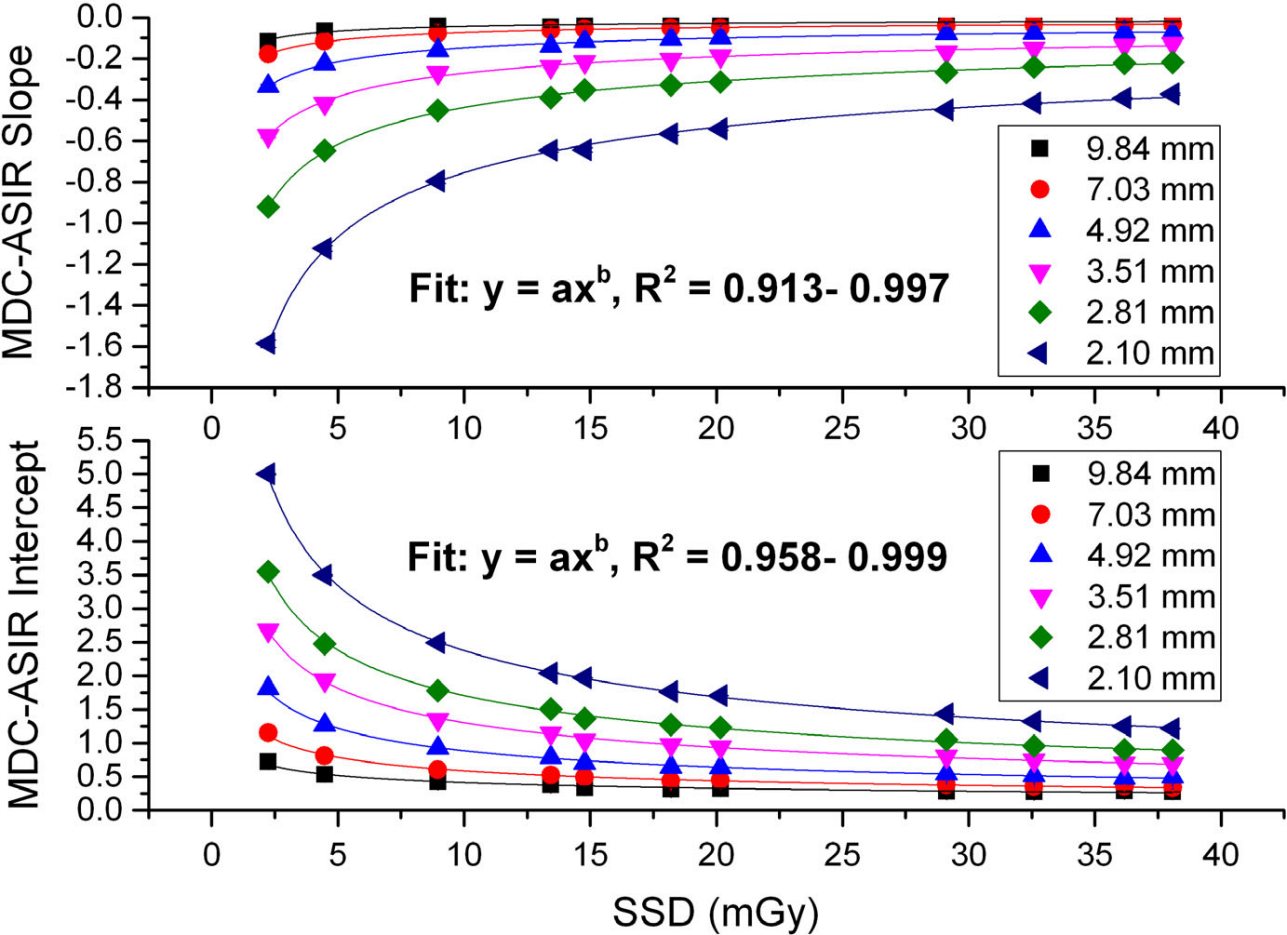
Minimum detectable contrast ‐ adaptive statistical iterative reconstruction fraction slope/intercept versus dose shows power law relationships.

**Figure 5:**
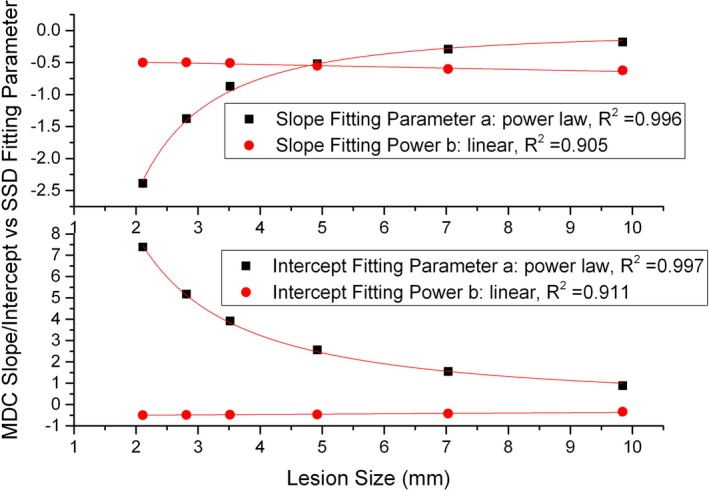
Power law relationship between the proportionality factor/power index in Fig. 4 and the lesion size.

The results of SD‐MDC versus ASIR fraction are shown in Fig. 3, where the SSDs were labeled. The linear relation is clearly seen, suggesting *q = 1* in Eq. (2) in Section 2.2. The correlation coefficients of the linear fit were found to be in the range of 0.985 to 0.998. The slope and intercept vary with the dose and the lesion size as indicated in Eq. (2). As demonstrated in the figure, both the slope magnitude and intercept decrease as the dose or target size increases. Their dependencies on the dose are shown in Fig. 4. It was found that the power‐law form in Eqs. (3) and (4) showed the best least‐square fit. The correlation coefficients ranged from 0.958 to 0.999, and from 0.913 to 0.997, for the intercept fit and the slope fit, respectively. The fitted proportionality factor and power index were related to the lesion size as shown in Fig. 5. The results in Figs. 4 and 5 can be integrated, and the terms in Eqs. (2), (3), (4) can be written as follows:

(5) q=1,

(6) $$a_{0}\left(d\right)=c_{1}d^{c_{2}},$$

(7) $$b_{0}\left(d\right)=c_{3}+c_{4}d,$$

(8) $$a_{\lambda }\left(d\right)=c_{5}d^{c_{6}},$$

(9) $$b_{\lambda }\left(d\right)=c_{7}+c_{8}d.$$

The fitting parameters (*c_1_* to *c_8_*) are given in Table 1. To investigate the accuracy of using Eq. (1) and the parameters in Table 1, the resulted MDCs were compared to the discrete MDCs obtained at the data points following the MDC definition in the experiment. The results are given in Table 2.

**Table 1:** Fitting parameters *c_1_* to *c_8_* and correlation coefficients (in parentheses) in Eqs. (6) to (9).

c_1_	c_2_	c_3_	c_4_	c_5_	c_6_	c_7_	c_8_
19.717	−1.302	−0.531	0.017	−8.829	−1.774	−0.456	−0.019
(0.911)		(0.997)		(0.905)		(0.996)	

**Table 2:** Standard error of estimates using Eq. (1) and the fitting parameters *c_1_* to *c_8_*.

Lesion size (mm)	Number of data points	Standard error of estimate (%)
9.84	106	7.15
7.03	10	4.24
4.92	106	3.04
3.51	106	2.20
2.81	106	2.17
2.10	106	1.27

### 3.4 Constraint on dose and ASIR blending fraction

**Figure 6:**
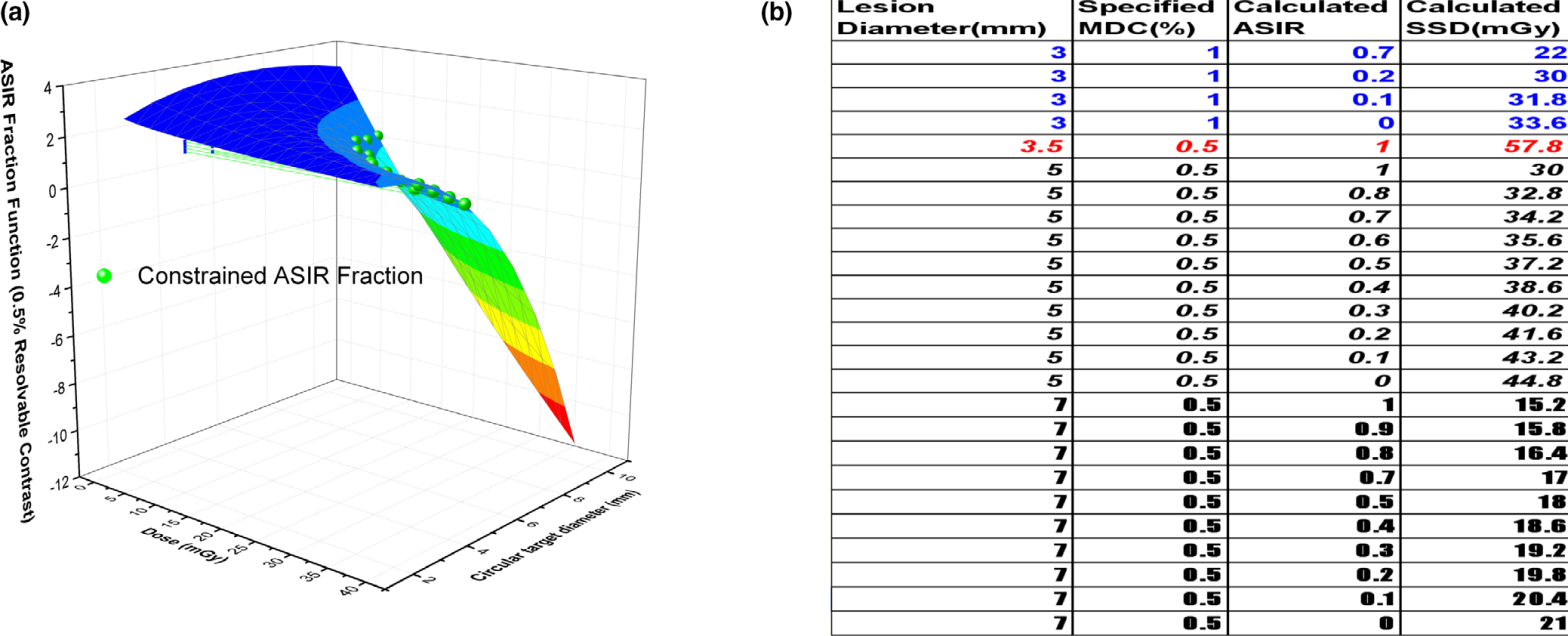
For the specified MDC = 0.5%, ASIR blending fraction *s* as a function of the dose and the target size with the sphere symbols representing the constrained choices (a), and an excerpt of the look‐up‐table for the ASIR fraction and dose with the given MDC (MDC accuracy = 0.001%, dose step = 0.2 mGy in the iteration) at certain diameters (b).

From Eqs. (2) and (5), the ASIR blending fraction *s* can be written as follows:

(10) $$s\left(\alpha ,D,d\right)=\frac{\alpha -a_{0}D^{b_{0}}}{a_{\lambda }D^{b_{\lambda }}},$$

where α is the MDC, *d* is the target size, and (*a_0,_ b_0_*, *a_λ_*, and *b_λ_*) are given by Eqs. (6)–(9) and Table 1. For a given α, *s* is a function of *D* and *d*. For instance, for α = 0.5%, the function in the relevant range of *D* and *d* is shown in Fig. 6(a), where circular diameter was used as the target dimension by considering area equivalence to a corresponding square. The spheres in the figure represent the constrained s by *0 < s ≤1*. Equation (10) can be used to build a look‐up‐table by excluding any ASIR blending fraction beyond the range of [0, 1]. Given the non‐linear feature of the dose in Eq. (10), a practical approach is to loop through various doses and ASIR blending fractions (from 0 to 1) until the calculated MDC reaches the specified MDC within the accuracy tolerance. An excerpt of the look‐up‐table is given in Fig. 6(b). Figure 6(b) demonstrates that, for the given MDC of the specified target size, the choices for *D* and* s* are restricted. For example, to resolve a lesion of 3.5 mm with 0.5% contrast (5 HU), the only dose choice is 57.8 mGy with 100% ASIR. However, to resolve a lesion of 3 mm with 1% contrast (10 HU), more distinct dose choices (say a difference bigger than 1 mGy) are available with various ASIR fractions (33.6 mGy at 0% ASIR to 22 mGy at 70% ASIR). For the lesion of 5 mm or 7 mm with 5% (5 HU) contrast, there are multiple dose choices with the corresponding ASIR fractions for either size. Obviously, the dose reduction rate also depends on the initial dose for the cases where multiple choices exist.

## 4 DISCUSSION AND CONCLUSION

In the presence of iterative reconstruction, the dose and noise relationship from the FBP is no longer valid. The lack of a quantitative relationship may lead to different conclusions on the viability of dose reduction. For task specific diagnosis, the required LCD can be defined first. Then the dose optimization task is reduced to whether multiple dose choices are available along with the iterative reconstruction blending fraction. This study presented a quantitative approach to determine the attainable dose and iterative reconstruction blending. As it was demonstrated, the existence of multiple dose choices depends on the specified LCD, quantified by the MDC at the concerned lesion size.

It is worthwhile to discuss the justification of a more general approach in Eq. (2) about the SD‐MDC’s dependency on the dose. If we define the normalized SD‐MDC (α_N_) as the ratio of the SD‐MDC with IR to the counterpart with FBP (α_N_ = α/α_0_) and use *q* = *1*, Eq. (2) can be written as follows:

(11) $$\alpha_{N}=1+\left(\frac{\lambda \left(D,d\right)}{\alpha_{0}\left(D,d\right)}\right)s.$$

**Figure 7:**
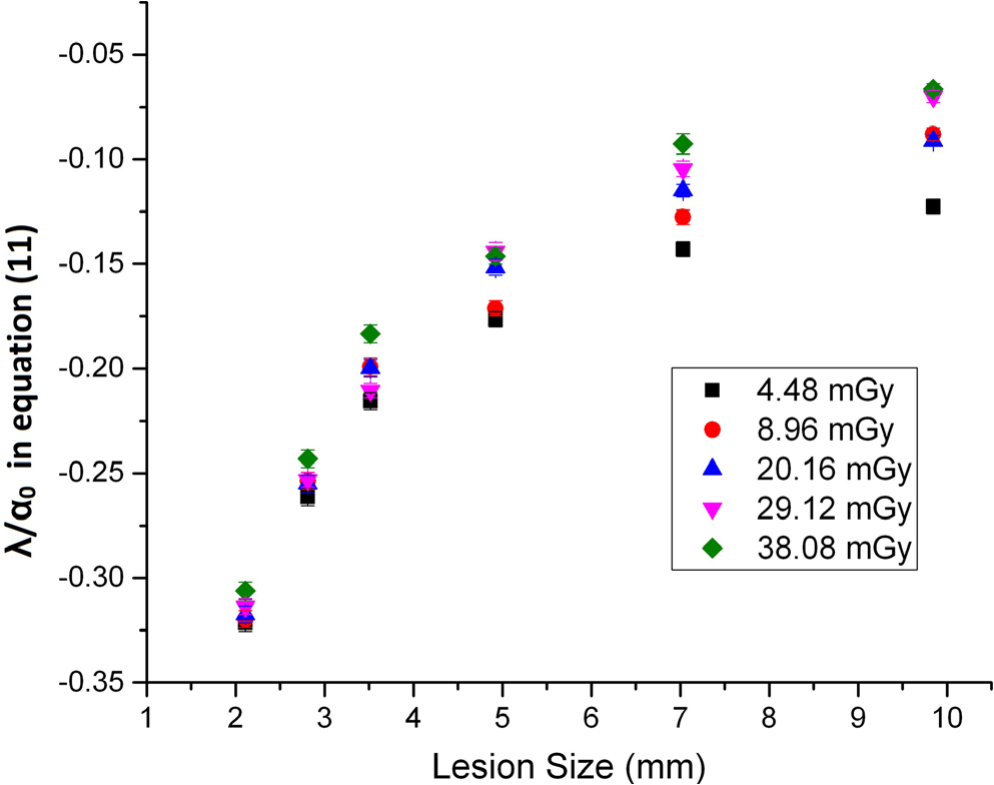
λ/α_0_ in Eq. (11) versus lesion size at various doses.

We previously utilized a simplified approach by assuming α_N_’s (therefore λ/α_0_) independence of the dose.^14^ It was then made straight forward to derive the relationship of SD‐MDC to the dose, target size, and ASIR blending fraction, as the SD‐MDC versus the dose with FBP was available from the work. However, validity of the assumption was only tested at two dose levels (8 mGy and 18 mGy). A more careful examination in a wider dose range was performed (SSD from 4.48 mGy to 38 mGy) in this study. Figure 7 shows λ/α_0_ in Eq. (11) as a function of the lesion size at different doses (SSD from 4.48 mGy to 38.08 mGy). It demonstrates that the relationship of the normalized MDC to the lesion size is in general dose dependent.

As a part of our study, it was found that the noises from the adjacent‐slices subtractions systematically underestimate the ground‐truth by 20%. This is due to the longitudinal correlation between different slices. The result does not depend on whether ASIR is utilized or not. This can be explained because the ASIR is slice based, it does not introduce additional inter‐slice correlation. Furthermore, the result is also pitch independent. It may be attributed to the manufacturer's pitch design optimization for the noise with helical scans.^17^ These findings can be useful for obtaining noises from clinical studies as repeated acquisitions are not practical.^18^

It was assumed in the study that the noise distribution is not altered in the presence of the signal. As the noise suppression may be signal dependent with iterative reconstructions (IR), this assumption may not be valid in general,^19^ especially with the model based IR. For ASIR, this aspect was visited in our previous work.^14^ Low contrast inserts were used in the tissue equivalent abdomen phantom. At two dose levels (8 mGy and 18 mGy), subtractions were made between the ASIR (0 – 100%) and FBP reconstructed images, and the noises were compared at various spatial scales (1.2 – 7 mm) between the background regions and the region with the contrast inserts. The comparisons showed that the noises in the low contrast region were signal independent. Since the result was a linear combination of the noises from FBP and from ASIR, the noises with ASIR were therefore signal independent. The study did not include a wider dose range; hence a further study may be necessary to draw a solid conclusion.

This study did not include the iterative reconstructions from different manufacturers. However, similar methodology is expected to be applicable if the granularity of the IR blending is made available.

The third‐generation iterative reconstruction ASIR‐V was not included in the study as it was not available on the machine. As ASIR‐V is not fully model based, it is expected that a similar approach to our study can be applied. It will be interesting to address the dose reduction availability quantitatively with ASIR‐V in our future work.

## CONFLICT OF INTEREST

The authors declare no conflict of interest.
